# Cloning and Characterization of a Putative R2R3 MYB Transcriptional Repressor of the Rosmarinic Acid Biosynthetic Pathway from *Salvia miltiorrhiza*

**DOI:** 10.1371/journal.pone.0073259

**Published:** 2013-09-10

**Authors:** Shuncang Zhang, Pengda Ma, Dongfeng Yang, Wenjing Li, Zongsuo Liang, Yan Liu, Fenghua Liu

**Affiliations:** 1 College of Life Sciences, Northwest A & F University, Yangling, People's Republic of China; 2 College of Life Sciences, Zhejiang Sci-Tech University, Hangzhou, People's Republic of China; 3 Tianjin Tasly Modern Traditional Chinese Medicine Resources Co., Ltd, Tianjin, People's Republic of China; University of Michigan, United States of America

## Abstract

*Salvia miltiorrhiza* Bunge is one of the most renowned traditional medicinal plants in China. Phenolic acids that are derived from the rosmarinic acid pathway, such as rosmarinic acid and salvianolic acid B, are important bioactive components in *S. miltiorrhiza*. Accumulations of these compounds have been reported to be induced by various elicitors, while little is known about transcription factors that function in their biosynthetic pathways. We cloned a subgroup 4 *R2R3* MYB transcription factor gene (*SmMYB39*) from *S. miltiorrhiza* and characterized its roles through overexpression and RNAi-mediated silencing. As the results showed, the content of 4-coumaric acid, rosmarinic acid, salvianolic acid B, salvianolic acid A and total phenolics was dramatically decreased in *SmMYB39*-overexpressing *S. miltiorrhiza* lines while being enhanced by folds in *SmMYB39*-RNAi lines. Quantitative real-time PCR and enzyme activities analyses showed that *SmMYB39* negatively regulated transcripts and enzyme activities of 4-hydroxylase (C4H) and tyrosine aminotransferase (TAT). These data suggest that *SmMYB39* is involved in regulation of rosmarinic acid pathway and acts as a repressor through suppressing transcripts of key enzyme genes.

## Introduction

*S. miltiorrhiza* Bunge, called ‘Dan-Shen’ in Chinese, is one of the most widely used traditional herbal medicines for the treatment of a variety of conditions, such as cardiovascular and cerebrovascular diseases [1]–[3], breast cancer [4] and hepatitis [5], [6]. The bioactive components of *S. miltiorrhiza* are divided into two groups, the water-soluble phenolic acids and the lipid-soluble tanshinones [7], [8]. The water-soluble phenolic acids mainly contain caffeic acid, danshensu ((r)-a,3,4-trihydroxybenzenepropanoic acid), 4-coumaric acid, *t*-cinnamic acid, rosmarinic acid (RA) and salvianolic acid B (SAB) [9], [10]. The lipid-soluble tanshinones include tanshinone I, tanshinone IIA, dihydrotanshinone I, cryptotanshinone and etc., which belong to a group of diterpenes with an abietane-type skeleton [11], [12]. In recent years, the water-soluble phenolic acids have attracted attention for their marked pharmacological activities coupled with their traditional use from herbs steeped in boiling water in China.

**Figure 1:**
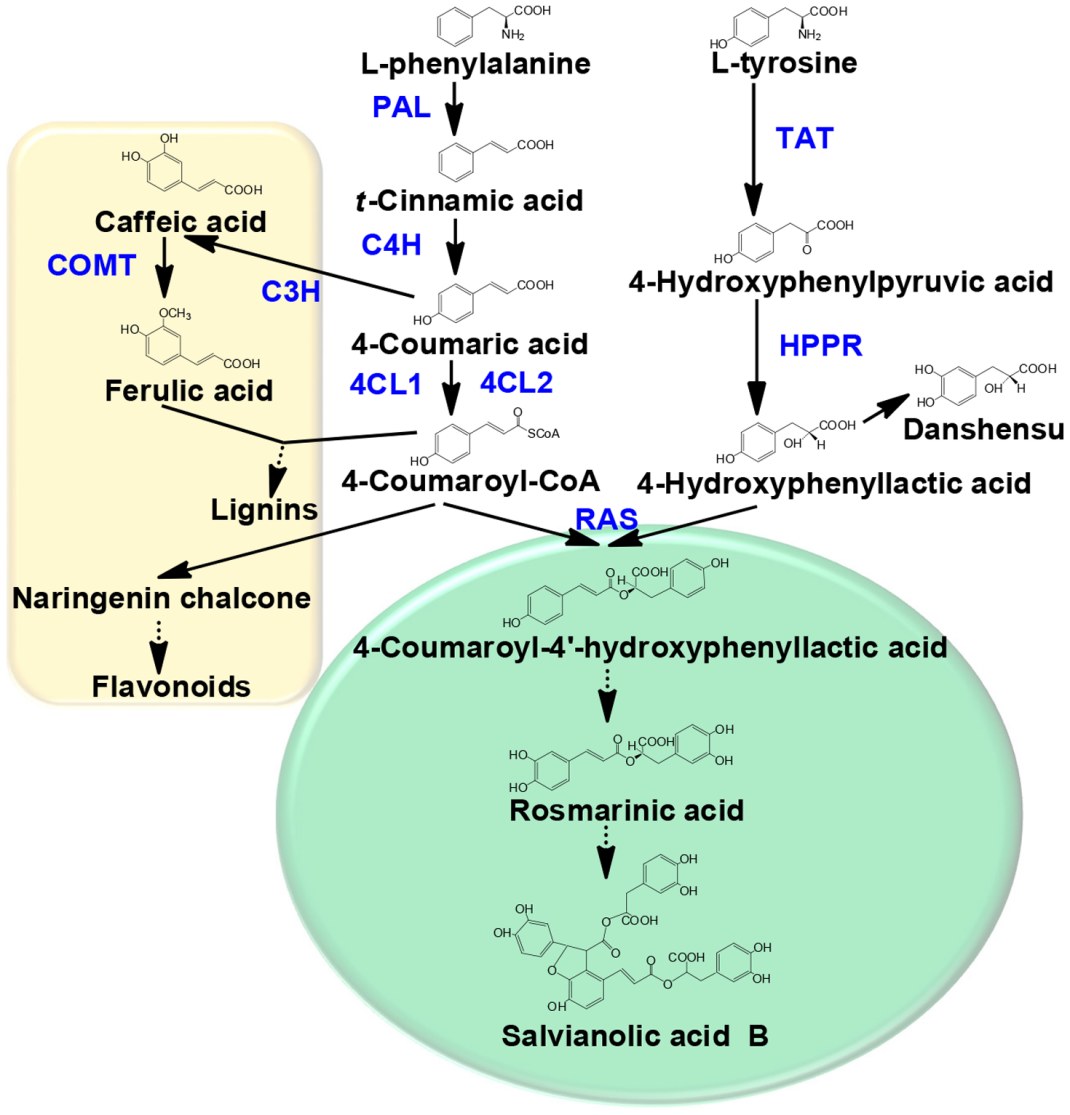
Phenolic acids biosynthetic pathway in *S. miltiorrhiza*. Multiple enzymatic steps are represented by dotted lines. The ‘circle’ and ‘square’ are used to distinguish between the downstream pathway of RA and other branches of phenylpropanes metabolism. C3H, coumarate 3-hydroxylase; C4H, cinnamic acid 4-hydroxylase; 4CL, 4-coumaric acid CoA-ligase; COMT, caffeic acid O-methyltransferase; HPPR, 4-hydroxyphenylpyruvate reductase; PAL, phenylalanine ammonia-lyase; RAS, rosmarinic acid synthase; TAT, tyrosine aminotransferase.

Water-soluble phenolic acids in *S. miltiorrhiza* are produced through the phenylpropanoid pathway and the biosynthetic pathway of RA is well characterized in plants. RA biosynthesis starts with the aromatic amino acids L-phenylalanine and L-tyrosine, which are separately converted to intermediate precursors 4-coumaroyl-CoA and 4-hydroxyphenyllactic acid through two parallel pathways. These two intermediate precursors are then covalently coupled by several biological reactions and generated RA [13], [14]. SAB is another important phenolic acid and an index chemical in the quality control of Dan-Shen [15]. It is deemed to be derived from RA, but the detailed pathway has not been characterized to date [16], [17]. Biosynthesis of most other phenolic acids are also closely related to RA pathway. For example, caffeic acid and ferulic acid are intermediate precursors of lignin biosynthetic pathway [18], which shares the upstream pathway with RA production. And *t*-cinnamic acid and 4-coumaric acid are the common intermediate precursors of many metabolites, such as RA, lignin and flavonoids (Fig. 1).

MYB proteins are one of the largest transcription factor families in plants. Among approximately 1700 transcription factor genes identified in the *Arabidopsis thaliana* genome, 339 belong to MYB members [19]. Based on the numbers of imperfect repeats (one, two, three or four) in the DNA-binding domain, MYB transcription factors are classified into four subfamilies and refered as 1R-MYB, 2R-MYB, 3R-MYB and 4R-MYB, respectively [20]. MYB proteins in plants are mainly 2R-MYB type, namely R2R3 MYBs, e.g. about 137 R2R3 members in *Arabidopsis* and 88 ones in rice [20]. The N-terminal DNA-binding domain (in the R2 and R3 repeats) of R2R3 MYB proteins is highly conserved; however the C-termininal amino acid sequences are various and have been considered to be responsible for their different regulating functions [21]. R2R3 MYBs have been categorised into 22 subgroups on the basis of conserved amino acid sequence motifs present C-terminal to the MYB domains [22], [23]. Amongst the 22 subgroups, members of subgroup 4 are suggested to act as transcriptional repressors of phenylpropanoid pathway and reduce production of phenylpropanoid metabolites by repressing transcripts of key enzymes. Two examples are *AmMYB308* and *AmMYB330* from *Antirrhinum majus*, which reduced contents of phenolic acids and lignins when over expressed in tobacco plants. Besides, overexpression of these two genes down-regulated several key enzyme genes in the phenylpropanoid pathway, such as 4-coumaric acid CoA-ligase (*4CL*), cinnamate-4-hydroxylase (*C4H*) and cinnamyl alcohol dehydrogenase (*CAD*) [24]. The *Arabidopsis AtMYB4* knock-out mutant exhibited an increase in the accumulation of sinapate esters and up-regulation of the *C4H* gene [25]. Overexpression of two subgroup 4 members from maize, *ZmMYB31* and *ZmMYB42*, in *A. thaliana* down-regulated both the *A. thaliana* and the maize caffeic acid O -methyl-transferase (*COMT*) genes and reduced lignin content in the transgenic plants [26], [27]. Overexpression of *FaMYB1* from strawberry severely repressed accumulation of cyanidin 3-rutinoside (an anthocyanin) and of quercetin-glycosides (flavonols) in flowers of transgenic tobacco lines [28]. *MdMYB6* from *Malus* × *domestica* led to a decreased anthocyanin production when over expressed in Arabidopsis [29].

Although biosynthetic pathway of RA has been characterized, little is known about transcription factors that function in this pathway. As the RA pathway shares the common phenylpropanoid pathway with other phenylpropanoid metabolites, it will be very interesting to investigate whether phenolic acids derived from the RA pathway can be regulated by subgroup 4 R2R3 MYB transcription factors. In this study, we isolated a gene of subgroup 4 R2R3 MYB member from *S. miltiorrhiza* and studied its roles in regulating the RA pathway through overexpression and RNAi-mediated silencing.

## Materials and Methods

### Plant materials

Mature seeds of *S. miltiorrhiza* were collected from Dan-shen cultivation base of Shaanxi Tasly plant medicine Co. Ltd. (Shangluo, P.R.China). They were used to get sterile plantlets as described by Yan and Wang [30]. Sterile plantlets were cultured on half strength Murashige and Skoog (MS) medium [31], supplemented with 30 g l^−1^ sucrose and 7 g l^−1^ agar. Leaves of these plantlets were used for the gene transformation. Two-year-old flowering *S. miltiorrhiza* were obtained from the medical plants garden of Northwest A&F University (May 14, 2011) and used for the analysis of tissue-specific expression pattern of *SmMYB39*. The field studies did not involve any endangered or protected species, and sample collection was authorized by the Agricultural Bureau of Shangluo and the management department of the medical plants garden of Northwest A&F University.

### Isolation of a subgroup 4 *R2R3 MYB* homolog from *S. miltiorrhiza*

Total RNA was isolated from sterile plantlets of *S. miltiorrhiza* using the RNAprep pure Plant Kit (TIANGEN, Beijing, China) and then reversely transcribed according to the manufacturer's instruction of PrimeScriptTM RT reagent Kit (Takara, Shiga, Japan) to generate cDNA. Degenerate primers were designed from conserved DNA-binding domains of subgroup 4 *R2R3 MYB* repressors in other plant species with sequences SmMYBP1: 5′-AGGTC(A/C/G/T)CC(G/T)TGCTG(C/T)GAGAA-3′ and SmMYBP2: 5′-G(A/G)CT(A/G)TG(C/G)AG(C/T)TTGATGATG-3′. The 50 µl PCR mixture contained 25 µl Premix Taq (Takara, Shiga, Japan), 2 µl first-strand cDNA and 0.4 µM each primer and the PCR reaction was performed as follows: pre-heating at 94°C for 5 min, 30 cycles at 94°C for 50 s, 55°C for 50 s and 72°C for 30 s, then an extension at 72°C for 10 min. The amplified fragments were subcloned into the pMD19-T vector (Takara, Shiga, Japan) and sequenced by Sangon Biotech Co., Ltd. (Shanghai, China)

Rapid amplification of cDNA ends (RACE) was performed to get full-length cDNAs of *R2R3 MYB* homologs under the manufacturer's instruction (Clontech, Palo Alto, CA, USA). Gene specific primers SmGSP1 (5′-TTGAGATCGGGGCGGAGGTAGTTGA-3′) and SmGSP2 (5′-ACAAACAAAGGGGCGTGGACTAAGGAAG-3′) were designed for 5′ RACE and 3′ RACE, respectively, based on the obtained conserved sequences. The PCR condition for 5′ RACE was as follows: 5 cycles at 94°C for 30 s, 72°C for 3 min; 5 cycles at 94°C for 30 s, 70°C for 30 s, 72°C for 3 min; 27 cycles at 94°C for 30 s, 65°C for 30 s, and 72°C for 3 min. The PCR condition for 3′ RACE consisted of 5 cycles at 94°C for 30 s, 72°C for 3 min; 5 cycles at 94°C for 30 s, 70°C for 30 s, 72°C for 3 min; 32 cycles at 94°C for 30 s, 68°C for 30 s, and 72°C for 3 min. The amplified fragments were subcloned into the pMD19-T vector and sequenced. The full-length cDNA sequence was assembled based on the 3′- and 5′- RACE sequences using the Lasergene 7.1 software (DNASTAR, Inc., Madison, USA). The assembled sequence was named *SmMYB39* and confirmed by PCR amplification and sequencing.

Genomic DNA was isolated from sterile plantlets of *S. miltiorrhiza* using the Genomic DNA Isolation Kit (Cowin Biotech, Beijing, China). Gene-specific primers SmMYBP3 (5′-ATGGGAAGGTCTCCTTGCTGTG-3′) and SmMYBP4 (5′-TCATTTCATCTCCAATCTTCTGTAA-3′) designed from the obtained full-length cDNA sequence were used to amplify the genomic clone of *SmMYB39*. Then the PCR products were cloned into pMD19-T vector and sequenced.

### Bioinformatics analysis

Self Optimized Prediction Method with Alignment (SOPMA, http://npsa-pbil.ibcp.fr/) was used to predict secondary structure of the deduced amino acids sequence of *SmMYB39*. SWISS-MODEL program (http://swissmodel.expasy.org/) was used to create a 3-D structural model of *SmMYB39* protein sequence based on the known crystal structure of a c-MYB from *Mus musculus* (name not assigned, PDB ID: 1H88, Chain ID: C) [32]–[34]. BLAST search is publicly available at the National Center for Biotechnology Information (NCBI) web site (www.ncbi.nlm.nih.gov/BLAST/), which was used to do homology search. Homologous R2R3 MYB protein sequences were used to perform a phylogenetic analysis using the PhyML method and tools available at Phylogeny.fr: (http://www.phylogeny.fr/) [35].

### Subcellular localization analysis

A vector pTF486 containing the open reading frame of *eGFP* was used in this study [36]. Total RNA was isolated from sterile plantlets of *S. miltiorrhiza* using the RNAprep pure Plant Kit (TIANGEN, Beijing, China) and then reversely transcribed according to the manufacturer's instruction of PrimeScriptTM RT reagent Kit (Takara, Shiga, Japan) to generate cDNA. The cDNA was used as the template for the subsequent RCR. The whole coding sequence of *SmMYB39* was amplified with primers SmGFPP1-*Sal* I (5′-ACGCGTCGACATGGGAAGGTCTCCTTGCTGTG-3′) and SmGFPP2-*Bam*H I (5′-CGCGGATCCACCACCACCACCACCTTTCATCTCCAATCTTCTGTAATCC-3′) using Pfu DNA Polymerase (Fermentas, Glen Burnie, USA). The amplification sequence was ligated with *Sal* I and *Bam*H I -digested pTF486 vector to generate a *SmMYB39*-*GFP* fusion construct under the control of cauliflower mosaic virus 35S (CaMV 35S) promoter. The construct was confirmed by sequencing and used for transient transformation of onion epidermis via a gene gun (Bio-Rad, Hercules, CA, USA). After 24 h of incubation, GFP fluorescence in transformed onion cells was observed under a confocal microscope (Nikon A 1, Tokyo, Japan).

### Construction of plant expression vectors and plant transformation

Total RNA was isolated from sterile plantlets of *S. miltiorrhiza* using the RNAprep pure Plant Kit (TIANGEN, Beijing, China) and then reversely transcribed according to the manufacturer's instruction of PrimeScriptTM RT reagent Kit (Takara, Shiga, Japan) to generate cDNA. The cDNA was then used as a template for the construction of plant expression vectors.

The whole coding sequence of *SmMYB39* was amplified with primers SmMYBP5-*Bam*H I (5′-CGCGGATCCATGGGAAGGTCTCCTTGCTGTG-3′) and SmMYBP6-*Bam*H I (5′-CGCGGATCCTCATTTCATCTCCAATCTTCTGTAA-3′) using Pfu DNA Polymerase (Fermentas, Glen Burnie, USA). The PCR products were cloned into pBluescript KS+ vector and then subcloned into the *Bam*H I -digested binary vector pBI111L [37]. The direction of the inserted sequence in the construct was identified by digesting with restriction enzymes and sequencing (see Fig. S1A).

For construction of the RNAi plasmid, a 259 bp fragment from the 3′ end of *SmMYB39* cDNA was PCR amplified using gene-specific primers (SmMYBP7-*Xho* I: 5′-CCGCTCGAGTGATCCCACTACGCATCGC-3′ and SmMYBP8-*Eco*R I: 5′-CCGGAATTCGGTATTTGTACAGCTGCAATCTTTG-3′; SmMYBP9- *Hind* III: 5′-CCCAAGCTTTGATCCCACTACGCATCGC-3′ and SmMYBP-*Cla* I: 5′-CCATCGATGGTATTTGTACAGCTGCAATCTTTG-3′) and ligated into the pKANNIBAL vector containing the pyruvate orthophosphate dikinase (PDK) intron [38] both in the sense and antisense orientations. An interfering box containing CaMV 35S promoter, the sense and antisense fragments on either side of the PDK intron and OCS terminator was excised from pKANNIBAL with *Not* I (Takara, Shiga, Japan) and cloned into the pART27 vector (see Fig. S1A) [39]. The recombinant plasmid was then identified by sequencing.

Constructs were introduced into *Agrobacterium tumefaciens* EHA105 by electroporation (Eppendorf Multiporator, Eppendorf, AG, Germany). Kanamycin-resistant colonies were verified by PCR-amplification, and PCR-positive colonies were used in the subsequent plant transformation. Transgenic plantlets were obtained as described by Song et al. [40]. A single clone of *A. tumefaciens* EHA105 harboring the *SmMYB39*-overexpression or *SmMYB39*-RNAi vector was inoculated into 10 ml liquid LB medium that contained 20 mg l^−1^ rifampicin and 50 mg l^−1^ kanamycin, and then grown on a shaker (180 rev. min^−1^) at 28°C for 16–18 h in the dark. Cells were collected by centrifugation (at 2292 g for 10 min) when the OD_600_ reached 0.6, and were re-suspended in 20–30 ml liquid MS medium. Sterile leaves were cut into 0.5×0.5 cm discs and pre-cultured for 1 day on the MS basal medium supplemented with 1.0 mg l^−1^ 6-Benzylaminopurine (6-BA) and 0.1 mg l^−1^ 1-naphthlcetic acid (NAA). Then the discs were submerged with shaking in the bacterial suspension for 25–30 min. Excess bacteria were later blotted, and the discs were transferred to the same media type and cultured for 3 days. They were then moved to a selection medium (MS basal medium supplemented with 1.0 mg l 6-BA, 0.1 mg l^−1^ NAA, 200 mg l^−1^ cefotaxime sodium and 50 mg l^−1^ kanamycin). After four cycles of selection (10 days each), the regenerated buds were transferred to the 1/2-strength MS basal medium supplemented with 30 mg l^−1^ kanamycin for root formation and elongation. Buds regenerated from leaf discs that had not been submerged in bacteria but were only cultured on the MS basal medium supplemented with 1.0 mg l^−1^ 6-BA and 0.1 mg l^−1^ NAA were used as the untransformed control. Rooted plantlets were cut from their internodes into segments and were cultured on the 1/2-strength MS basal medium for propagation. Two- month-old plantlets were used for analysis.

### Identification of transgenic plantlets by PCR

DNA was isolated according to the manufacturer's instruction of Genomic DNA Isolation Kit (Cowin Biotech, Beijing, China) from two-month-old kanamycin-resistant plantlets and used as template in PCR analysis for detecting transgenic lines (see Fig. S1B). Two pair primers, Sm35S1F (5′-GATTGATGTGATATCTCCACTGACG-3′) and SmMYB39-11R (5′-GAGACTACAAGCGAAGCACAAGG-3′), NPTII-1F (5′-GCTATGACTGGGCACAACAGACAAT-3′) and NPTII-1R (5′-GCGATACCGTAAAGCACGAGGA-3′), were designed for *SmMYB39*-overexpressing lines identification. Sm35S1F located at the 35S promoter of pBI111L vector and SmMYB39-11R was designed from the *SmMYB39* gene. Both NPTII-1F and NPTII-1R located at the neomycin phosphotransferase II (*NPTII*) gene of pBI111L vector (see Fig. S1A). Primers used for the selection of *SmMYB39*-RNAi lines were Sm35S2F (5′-AAGGACAGTAGAAAAGGAAGGTGGC-3′) and SmMYB39-22R (5′-ATAGGGAGGGCTGATTCTGAGGTC-3′), NPTII-2F (5′-CCGCAACTTCTTTACCTATTTCCG-3′) and NPTII-2R (5′-CGATACCGTAAAGCACGAGGAA-3′). Sm35S2F located at the 35S promoter of the interfering box and SmMYB39-22R was designed from the 259 bp fragment of *SmMYB39*. NPTII-2F and NPTII-2R were designed from the *NPTII* gene of pART27 vector (see Fig. S1A). All PCR products were verified by sequencing.

### Analysis of phenolic acids contents by HPLC

Compound extraction and analysis followed the methods described by Liang et al. [41] with minor modifications. The dried plantlet was ground to powder with mortar and pestle and sieved through a 0.45-mm screen. The sample powder (50 mg) was extracted with 10 ml 70% methanol under sonication for 45 min, and then centrifuged at 13201 g for 10 min. The supernatant was diluted with 70% methanol to 10 ml total volume and filtered through a 0.22 µm microporous membrane (Jinteng, Tianjin, China) before analysis. Contents of phenolic acids were determined by a Waters HPLC system (Waters, Milford, MA, USA) equipped with a 1525 binary pump, a manual sample injector, and a Waters 2996 photodiode array detector. Chromatography separation was performed with a C18 column (Waters, SunFire C18, 4.6 mm×250 mm, 5 µm particle size) at 30°C with a sample injection volume of 20 µl. Empower 2 software was used for data acquisition and processing. Detection at 280 nm using a flow rate of 1.0 ml min^−1^ over a gradient of acetonitrile (Fisher Scientific, Springfield, NJ, USA) (buffer A) against 0.02% phosphoric acid (Kermel, Tianjin, China) solution (buffer B) was used as follows: 0–10 min, 5–20% A (v/v); 10–15 min, 20–25% A (v/v); 15–20 min, 25% A (v/v); 20–25 min, 25–20% A (v/v); 25–28 min, 20–30% A (v/v); 28–36 min, 30% A (v/v); 37–44 min, 100% A (v/v). Standards of phenolic acids were purchased from the National Institute for the Control of Pharmaceutical and Biological Products (Beijing, China).

### Analysis of total phenolics

The extraction and analysis of total phenolics followed methods reported by Yan et al. [42]. The dried plantlet was ground to powder with mortar and pestle and 100 mg sample powder was extracted with 15 ml phosphate buffer (75 mM, adjusted to pH 7.0 with NaOH) by vortexing for 1 min. The mixture was centrifuged at 13201 g for 20 min. The supernatant was collected as total phenolic extract, which was subsequently diluted with the extraction buffer into a suitable concentration for analysis. Total phenolic content was determined with Folin-Ciocalteu reagent (Sigma-Aldrich, St. Louis, MO, USA) using gallic acid as standard. The extract solution (20 µl) was mixed in a test tube with 1.58 ml distilled water and 100 µl Folin-Ciocalteu reagent for 8 min, and then incubated with 300 µl sodium carbonate solution at 40°C for 30 min. The absorbance was measured at 765 nm against a reagent blank without the extract.

### Expression analysis by quantitative real-time PCR (qRT-PCR)

Total RNA was isolated from transgenic and control plantlets using the RNAprep pure Plant Kit (TIANGEN, Beijing, China) and then reversely transcribed according to the manufacturer's instruction of PrimeScriptTM RT reagent Kit (Takara, Shiga, Japan) to generate cDNA. The obtained cDNA was used as template for the qRT-PCR analysis. Primers were designed as described in Table 1 to detection expression levels of *SmMYB39*, phenylalanine ammonia lyase (*PAL*), cinnamic acid 4-hydroxylase (*C4H*), 4-coumaric acid CoA-ligase (*4CL1* and *4CL2*), tyrosine aminotransferase (*TAT*) and hydroxyphenylpyruvate reductase (*HPPR*). The constitutively expressed *actin* gene was used as an internal control. QRT-PCR was performed according to the manufacturer's instruction (Takara, Shiga, Japan) under the following condition: 30-s pre-denaturation at 95°C, 1 cycle; 5-s denaturation at 95°C, 30-s annealing using calculated Tm, 5-s collection fluorescence from 65°C to 95°C, 40 cycles. Quantification of the gene expression was done with comparative Ct method. Experiments were performed in triplicate, and the results were represented by their means ± SD.

**Table 1:** Primers used for the quantitative real-time PCR analysis.

Gene	Accession	Forward (5′–3′)	Revere (5′–3′)
*SmMYB39*	KC213793	GCCCTCCCTATCAACAAGAAC	ATCCCAGAAAATCGAATCCAG
*PAL*	DQ408636.1	ACCTACCTCGTCGCCCTATGC	CCACGCGGATCAAGTCCTTCT
*C4H*	DQ355979.1	CCAGGAGTCCAAATAACAGAGCC	GAGCCACCAAGCGTTCACCAA
*4CL1*	AY237163.1	ATTCGCATTCGCATTTCTCGG	GCGGCGTAGTGCTTCACCTTT
*4CL2*	AY237164.1	CGCCAAATACGACCTTTCCTC	TGCTTCAGTCATCCCATACCC
*TAT*	DQ334606.1	TTCAACGGCTACGCTCCAACT	AAACGGACAATGCTATCTCAAT
*HPPR*	DQ099741.1	GACTCCAGAAACAACCCACATT	CCCAGACGACCCTCCACAAGA
*Actin*	HM231319.1	GGTGCCCTGAGGTCCTGTT	AGGAACCACCGATCCAGACA

### Analysis of C4H and TAT activities

C4H activity was measured as described by Lamb and Rubery with some modifications [43]. The enzyme extract was added to 4.8 ml reaction buffer (50 mM phosphate buffer containing 2 mM 2-mercaptoethanol, 2 mM *t*-cinnamic acid and 0.5 mM NADPH) and then incubated for 30 min at 30°C. The reaction was stopped with 6 M HCl and readjusted to pH 11 with 6 M NaOH. Absorbance value of the sample was measured at 310 nm.

TAT activity was determined using the method reported by Yan et al. [42]. The reaction mixture consisted of 200 µl enzyme extract, 200 µl L-tyrosine (88 mM), 200 µl α-ketoglutarate (10 mM), 100 µl pyridoxal phosphate (0.2 mM) and 3 ml kalium phosphate buffer at pH 7.5. The mixture was incubated at 37°C for 30 min and reaction was stopped with 1 ml 10 M NaOH. Initial (0 min) and final absorbance (30 min) of the reaction solution against a reagent blank at 331 nm were recorded.

### Statistical analysis

Statistical analyses were carried out with SPSS software (version 18.0, SPSS, Inc, Chicago, IL, USA). Spearman's correlation was used to assess the relation between the expression levels of *SmMYB39* and total phenolics content in different tissues of two-year-old flowering *S. miltiorrhiza*. ANOVA was used to identify metabolites accumulation, gene transcripts and enzyme activities that showed significant (*P*<0.05) changes in relative abundance in the transgenic *S. miltiorrhiza* lines.

## Results

### Isolation of *SmMYB39* and sequence analysis

**Figure 2:**
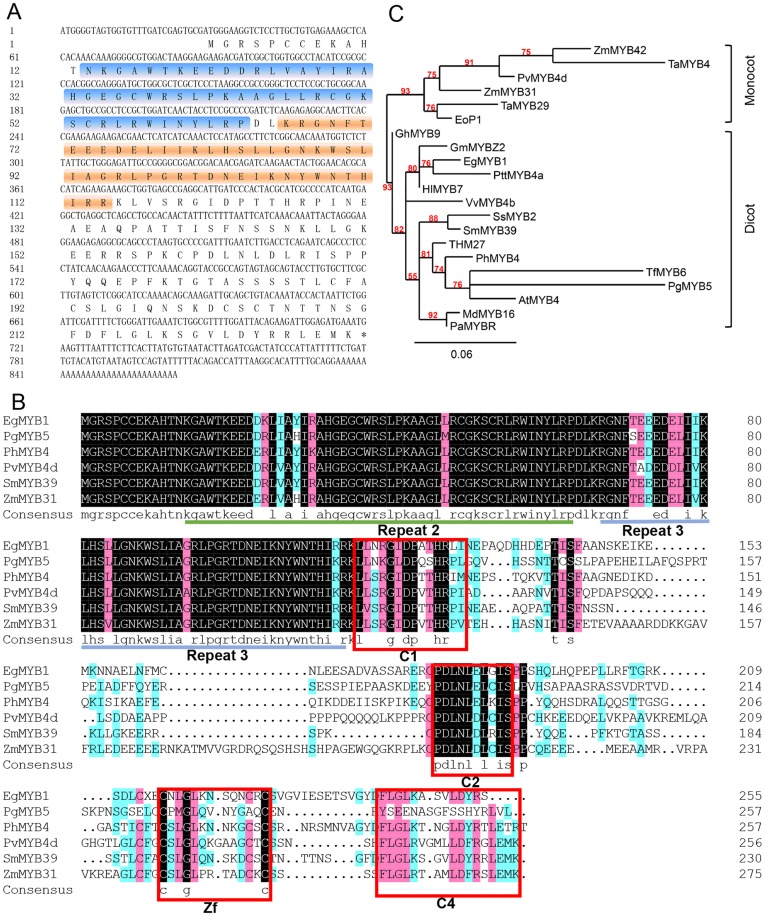
Sequence analysis of SmMYB39. (A) Nucleotide sequence of *SmMYB39* with amino acid translation. R2 and R3 repeats are highlighted in blue and orange, respectively. (B) Amino acid alignment of SmMYB39 with known R2R3-MYB regulators of phenylpropanes metabolism from other species. R2 and R3 repeats are underlined. The boxed sequences are the potential functional motifs. (C) Phylogenetic tree of SmMYB39 and known R2R3 MYB transcription factors from other plant species. Accession numbers of the proteins in the GenBank database are as follows: ZmMYB42 (NP_001106009.1, *Zea mays*), TaMYB4 (AEG64799.1, *Triticum aestivum*), PvMYB4d (AEM17351.1, *Panicum virgatum*), ZmMYB31 (NP_001105949.1, *Zea mays*), TaMYB29 (AEV91152.1, *Triticum aestivum*), EoP1 (ADL18407.1, *Eremochloa ophiuroides*), GhMYB9 (AAK19619.1, *Gossypium hirsutum*), GmMYBZ2 (NP_001235092.1, *Glycine max*), EgMYB1 (CAE09058.1, *Eucalyptus gunnii*), PttMYB4a (CAD98762.1, *Populus tremula* × *Populus tremuloides*), HlMYB7 (CCC14990.1, *Humulus lupulus*), VvMYB4b (ACN94269.1, *Vitis vinifera*), SsMYB2 (ABP57083.1, *Solenostemon scutellarioides*), THM27 (NP_001233975.1, *Solanum lycopersicum*), PhMYB4 (ADX33331.1, *Petunia* × *hybrida*), TfMYB6 (AAS19480.1, *Tradescantia fluminensis*), PgMYB5 (ABQ51221.1, *Picea glauca*), AtMYB4 (NP_195574.1, *Arabidopsis thaliana*), MdMYB16 (ADL36756.1, *Malus* × *domestica*), PaMYBR (ADY15315.1, *Prunus avium*).

A 243-bp band was amplified with the degenerate primers. After being subcloned into pMD19-T vector, a total of six independent clones of the PCR products were sequenced; all sequences were identical and yielded a single full-length cDNA sequence (termed as *SmMYB39*) through RACE. *SmMYB39* (Genbank accession number: KC213793) contains an open reading frame (ORF) of 693 bp in length and encodes a 25.97-kDa protein (Fig. 2A). The protein sequences alignment between SmMYB39 and five known subgroup 4 R2R3 MYB transcription factors revealed the presence of four protein motifs such as C1 (LlsrGIDPxT/SHRxI/L), C2 (pdLNLD/ELxiG/S), Zf (CX_1-2_CX_7-12_CX_2_C) and C4 (FLGLX4-7V/LLD/GF/YR/SX1LEMK) (Fig. 2B). They were considered as conserved protein motifs of subgroup 4 R2R3 MYBs and suggested to be essential for these proteins to act as transcriptional regulators [22], [26], [44], [45].

A phylogenetic tree was constructed by the PhyML method based on an alignment of 21 R2R3 MYB protein sequences (Fig. 2C). According to the phylogenetic tree, all proteins were classified into two groups belonging to dicot and monocot plants. SmMYB39 is most closely related to SsMYB2 from Solenostemon scutellarioides, and the identity between the two amino acid sequences is 77%. It is rational as S. miltiorrhiza and S. scutellarioides belong to the same plant family (Lamiaceae). The known negative regulators of phenylpropanes metabolism, such as ZmMYB42, EgMYB1, PhMYB4 and AtMYB4, dispersedly distribute in the phylogenetic tree, suggesting that the functions of subgroup 4 R2R3 MYBs are conserved in different plant species.

The genomic sequence of *SmMYB39* (Genbank accession number: KC771280) was PCR amplified from genomic DNA using gene-specific primers. Comparison of the genomic DNA and cDNA sequences revealed that *SmMYB39* harbored a 69 bp intron in the R3 domain (see Fig. S2A). The secondary structure and 3-D structural model of SmMYB39 were predicted (see Text S1 and Fig. S2B, C).

### SmMYB39 is located in nucleus

**Figure 3:**
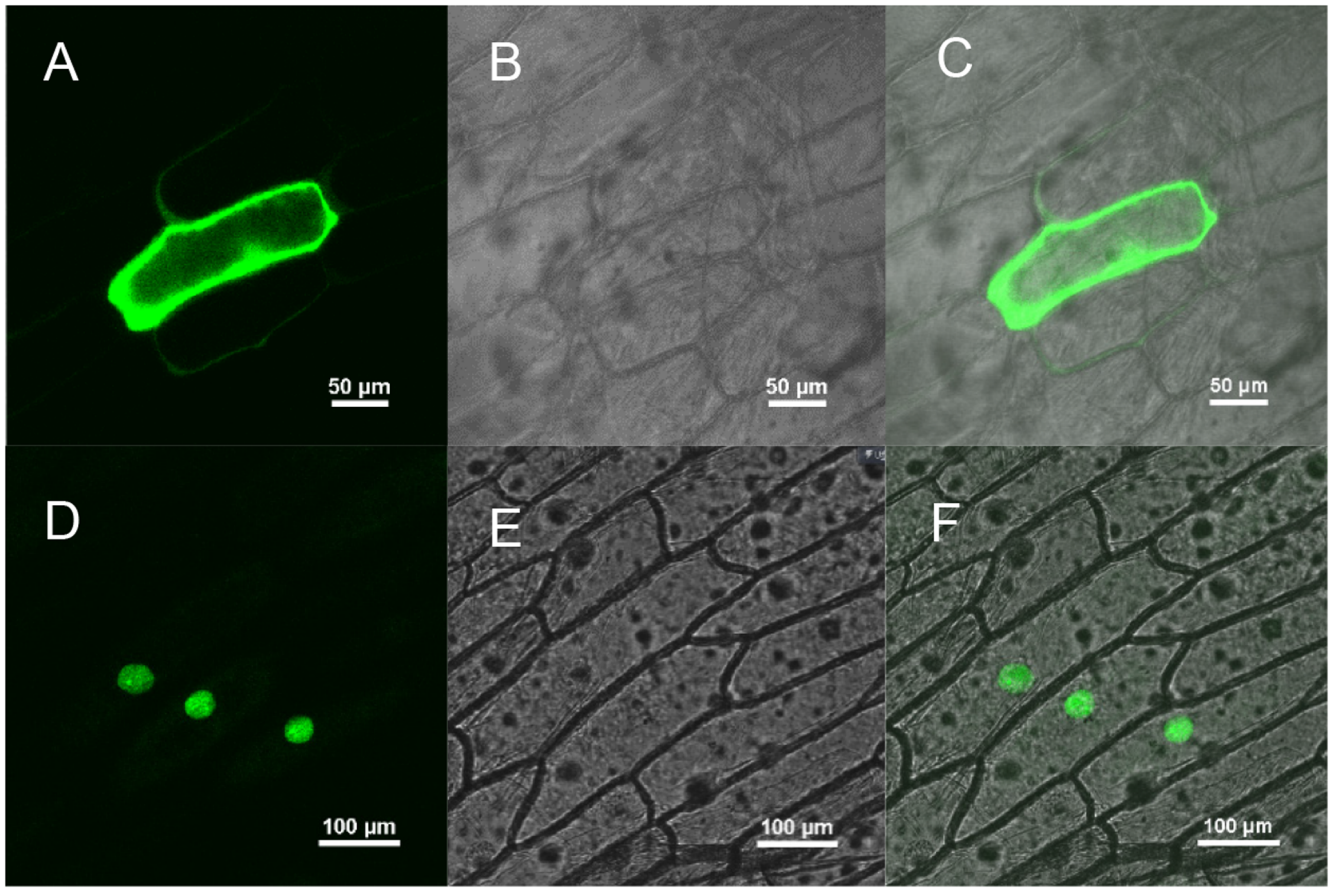
Nuclear localization of SmMYB39. Confocal images of onion epidermis cells under the GFP channel show the constitutive localization of GFP (A) and nuclear localization of SmMYB39 -GFP (D). The confocal images (B) and (E) are of the same cells in (A) and (D) with transmitted light, respectively. The image (C) is the merged image of image (A) and (B), and the image (F) is the merged image of image (D) and (E).

To examine the subcellular localization of SmMYB39, the open reading frame of *SmMYB39* was fused to the 5′ -terminus of the *GFP* reporter gene under control of the CaMV 35S promoter. The recombinant constructs of the *SmMYB39*-*GFP* fusion gene and *GFP* alone were introduced into onion epidermal cells by particle bombardment, respectively. As showed in Fig. 3, the SmMYB39-GFP fusion protein was specifically localized in the nucleus, whereas GFP alone showed ubiquitous distribution in the whole cell. This result indicated that the SmMYB39 protein was localized in the nucleus and may act as a transcription factor in gene transcriptional regulating.

### Tissue-specific expression pattern of *SmMYB39* in *S. miltiorrhiza*

**Figure 4:**
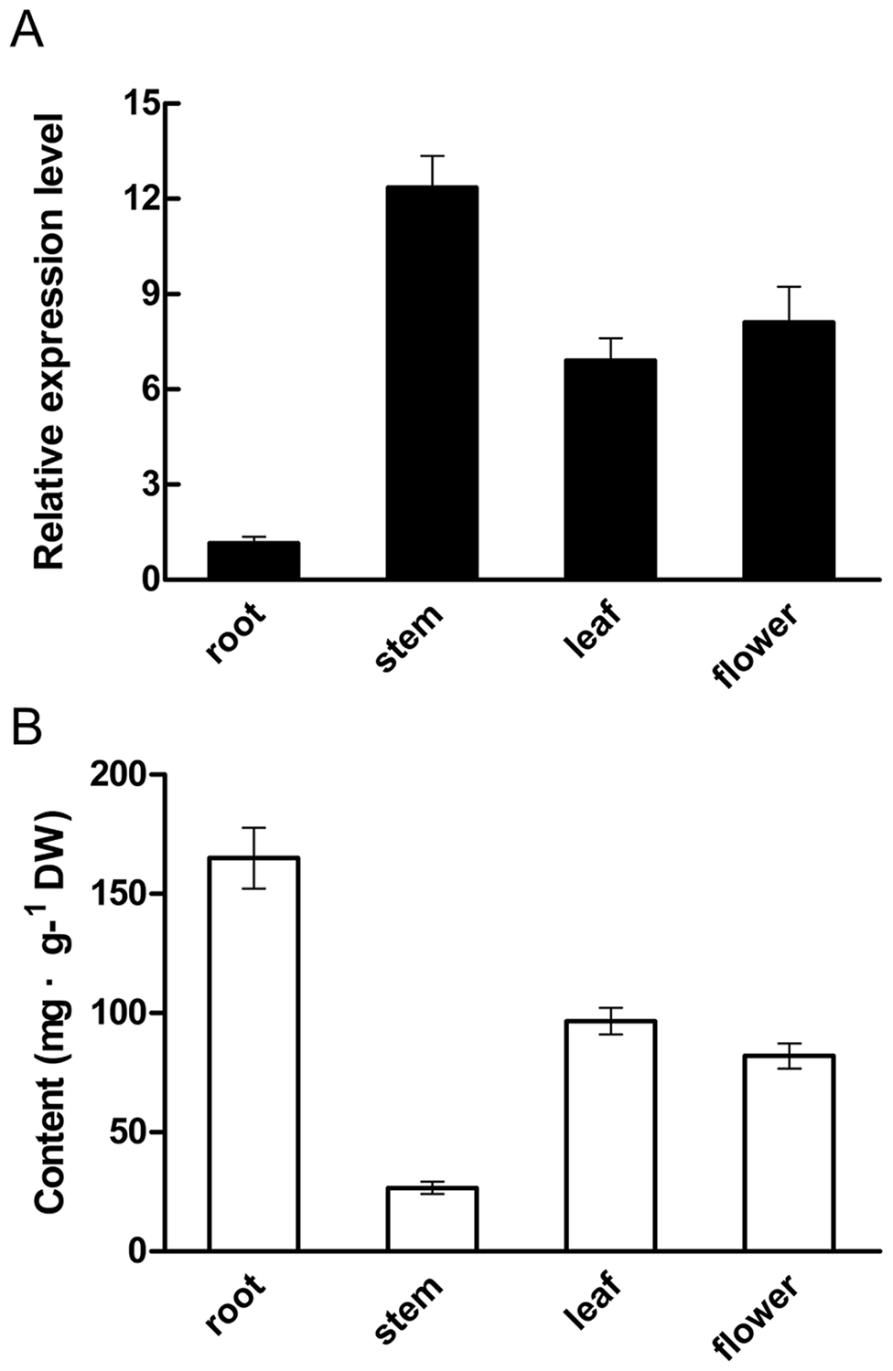
Relative expression levels of *SmMYB39* (A) and total phenolics content (B) in different tissues of *S. miltiorrhiza*. The results were analyzed using the comparative Ct method and presented as fold-changes compared with the root. The *S. miltiorrhiza actin* gene was used as an internal control to normalize expression levels. Data presented here are the mean of three replicates with error bars indicating ± SD.

QRT-PCR was performed to determine expression levels of *SmMYB39* in different tissues (root, stem, leaf and flower) of two-year-old flowering *S. miltiorrhiza*. Results showed that *SmMYB39* expressed in all the tissues tested, with the highest expression in stem and lowest expression in root. Total phenolics accumulation in corresponding tissues was also measured. In contrast to *SmMYB39* expression, the accumulation in root was the most while in stem was the least (Fig. 4). As the result of Spearman correlation analysis, *SmMYB39* expression was negative-correlated with accumulation of total phenolics (r = −0.916, *P*<0.01), implying that *SmMYB39* may be a repressor of phenolic acids biosynthesis in *S. miltiorrhiza*.

### *SmMYB39* suppresses phenolic acids biosynthesis in *S. miltiorrhiza*

**Figure 5:**
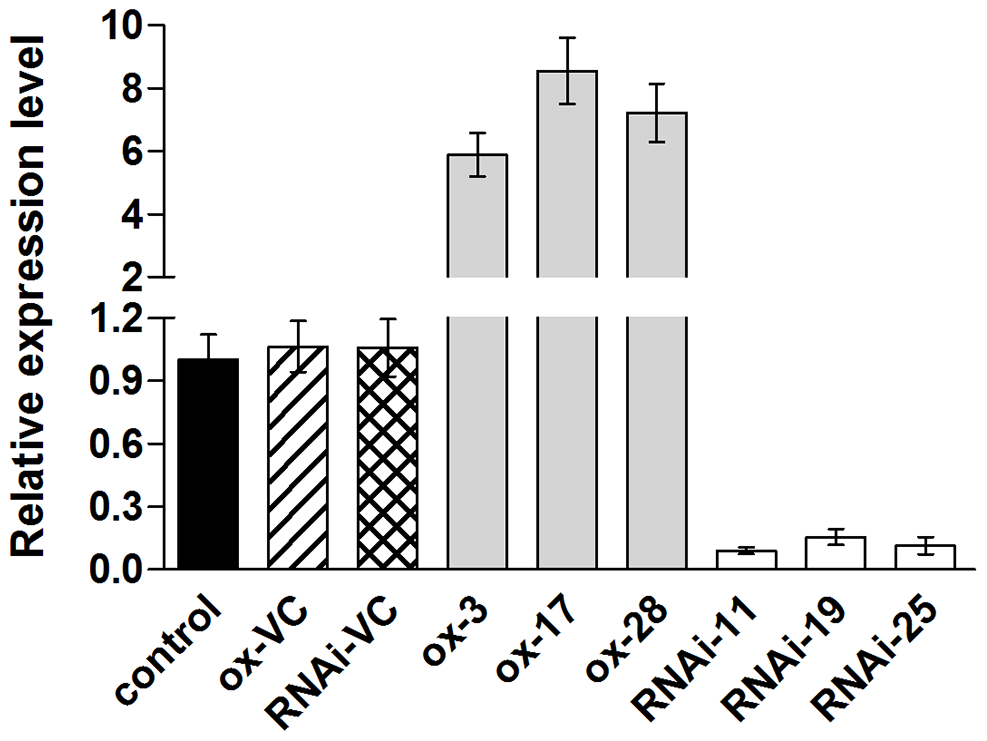
Relative quantitative analysis of *SmMYB39* expression in transgenic lines and controls of *S. miltiorrhiza*. The results were analyzed using the comparative Ct method and presented as fold-changes compared with the control sample (untransformed control). The *S. miltiorrhiza actin* gene was used as an internal control to normalize expression levels. control: untransformed plant; ox-VC, RNAi-VC: empty vector controls of *SmMYB39*-overexpressing lines and *SmMYB39*-RNAi lines; ox-3, ox-17, ox-28: *SmMYB39*-overexpressing lines; RNAi-11, RNAi-19, RNAi-25: *SmMYB39*-RNAi lines.

**Figure 6:**
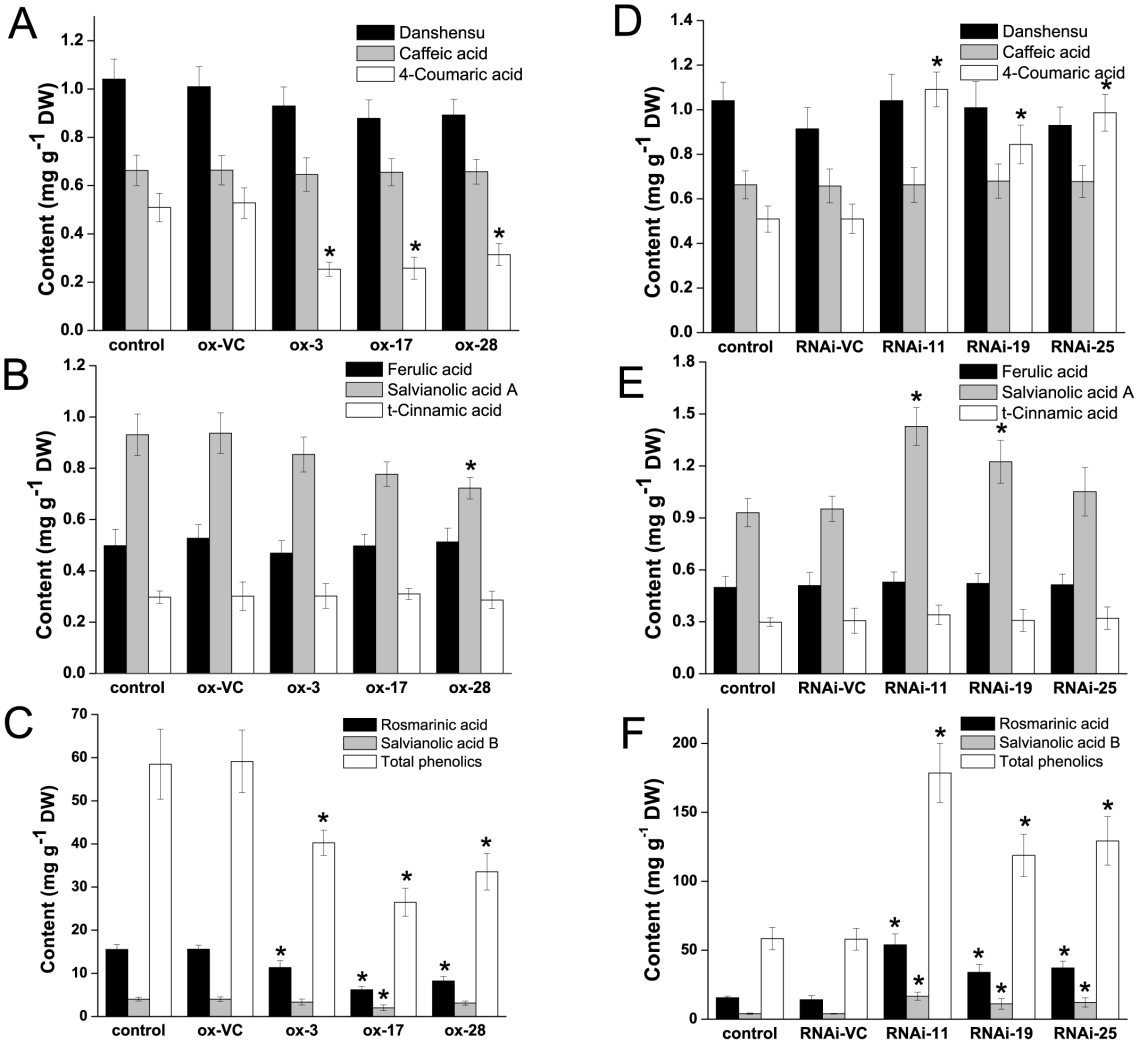
Analysis of related phenolic compounds contents in transgenic lines and controls of *S. miltiorrhiza*. Data presented here are the mean of three replicates with error bars indicating ± SD. The asterisks indicate statistically significant differences (*P*<*0.05*) compared to the empty vector control. control: untransformed plant; ox-VC, RNAi-VC: empty vector controls of *SmMYB39*-overexpressing lines and *SmMYB39*-RNAi lines; ox-3, ox-17, ox-28: *SmMYB39*-overexpressing lines; RNAi-11, RNAi-19, RNAi-25: *SmMYB39*-RNAi lines.

In this study, 31 transgenic *S. miltiorrhiza* plantlets overexpressing *SmMYB39* and 25 independent *SmMYB39*-RNAi lines were obtained. Three overexpressing lines (ox-3, ox-17 and ox-28) and three RNAi lines (RNAi-11, RNAi-19 and RNAi-25) that presented obvious changes in phenolic acids content were selected for analysis. Expression levels of *SmMYB39* in these six transgenic lines and controls were measured. The qRT-PCR results showed that the expression of the target gene was successfully regulated through genetic manipulation. Transcripts of *SmMYB39* in the three *SmMYB39*-overexpressing lines were approximately 5.53-, 8.04- and 6.80-fold with respect to the empty-vector control (the line that only transformed with empty expression vector), respectively. While in the three *SmMYB39*-RNAi lines, expression levels of *SmMYB39* only reached about 8.55%, 14.71% and 10.81% relative to the empty-vector control, respectively (Fig. 5). The contents of metabolites involved in the RA biosynthetic pathway were determined and the results revealed a broad reconfiguration of metabolic flux in transgenic plantlets. Compared with the untransformed plantlets and vector-control lines, accumulations of phenolic acids were dramatically (*P*<0.05) reduced in *SmMYB39*-overexpressing lines. For example, the content of 4-coumaric acid, rosmarinic acid, salvianolic acid B, salvianolic acid A and total phenolics were maximally decreased by 52.00% (in ox-3), 60.35% (in ox-17), 49.83% (in ox-17), 22.87% (in ox-28) and 55.21% (in ox-17), respectively (Fig. 6A, B, C). While in the transgenic *SmMYB39*-RNAi lines, production of the target metabolites was markedly (*P*<0.05) up-regulated. For instance, the content of 4-coumaric acid, rosmarinic acid, salvianolic acid B, salvianolic acid A and total phenolics in RNAi-11 line approximately reached 2.14-, 3.81-, 4.23-, 1.50-, and 3.08-fold of vector-control lines, respectively (Fig. 6D, E, F). Accumulations of other four tested phenolic acids, Danshensu, caffeic acid, ferulic acid and *t*-cinnamic acid were not affected by the genetic manipulation. Changes of metabolites content in transgenic lines implied that *SmMYB39* was involved in the RA biosynthetic pathway and acted as a repressor of phenolic acids production.

### *SmMYB39* down-regulates expression of *C4H* and *TAT* in RA pathway

**Figure 7:**
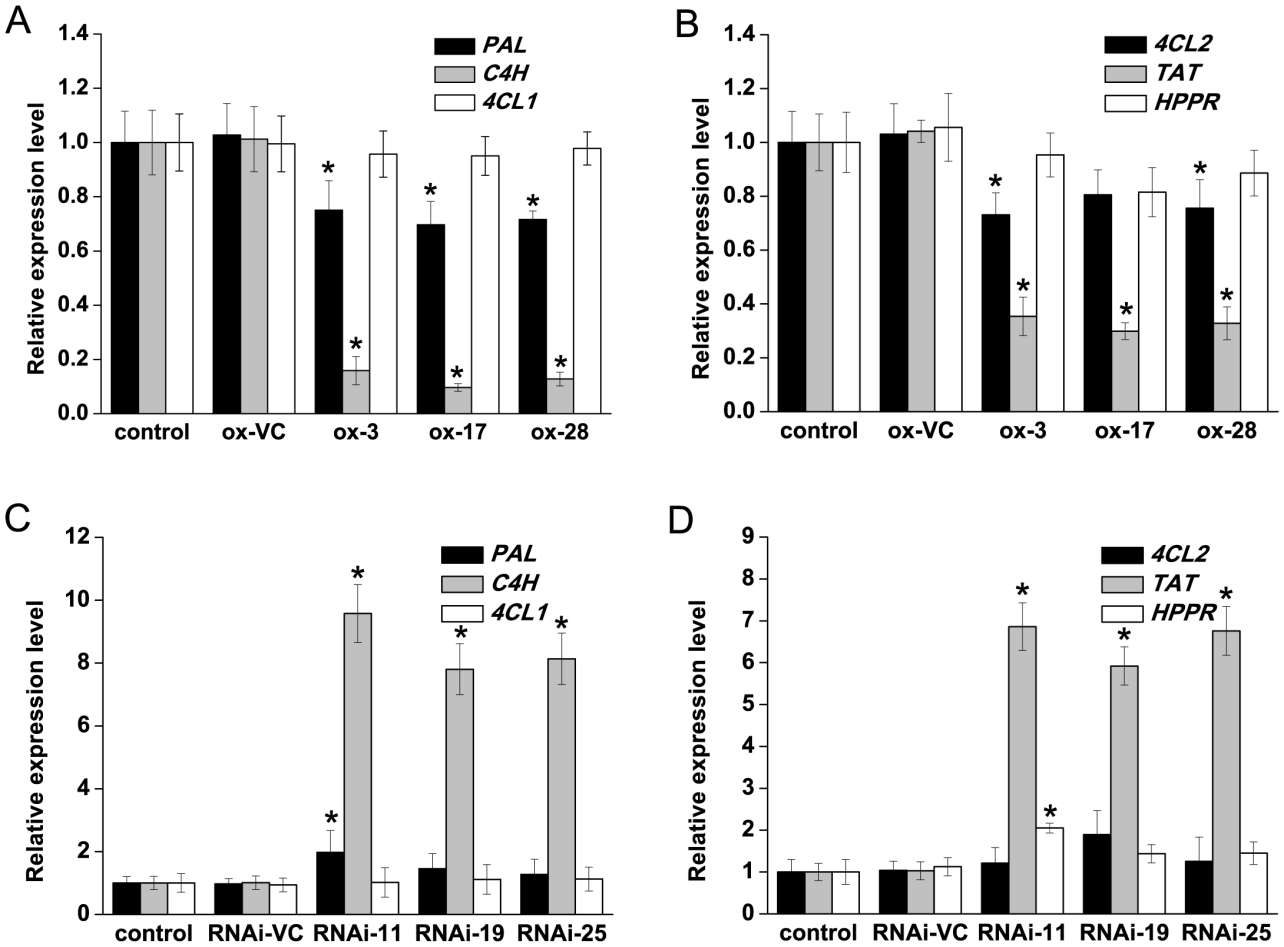
Relative quantitative transcripts analysis of enzyme genes in transgenic lines and controls of *S. miltiorrhiza*. The results were analyzed using the comparative Ct method and presented as fold-changes compared with the control sample (untransformed control). The *S. miltiorrhiza actin* gene was used as an internal control to normalize expression levels. The asterisks indicate statistically significant differences (*P*<*0.05*) compared to the empty vector control. control: untransformed plant; ox-VC, RNAi-VC: empty vector controls of *SmMYB39*-overexpressing lines and *SmMYB39*-RNAi lines; ox-3, ox-17, ox-28: *SmMYB39*-overexpressing lines; RNAi-11, RNAi-19, RNAi-25: *SmMYB39*-RNAi lines; *PAL*: phenylalanine ammonia-lyase; *C4H*: cinnamic acid 4-hydroxylase; *4CL*: 4-coumaric acid CoA-ligase; *TAT*: tyrosine aminotransferase; *HPPR*: 4-hydroxyphenylpyruvate reductase.

The qRT-PCR analysis of six key enzyme genes (*PAL*, *C4H*, *4CL1*, *4CL2*, *TAT*, and *HPPR*) in RA pathway was carried out in transgenic and untransformed plantlets. The results revealed that overexpression of *SmMYB39* down-regulated all the six genes at different levels. Compared with the empty-vector control lines, expression levels of *C4H* were reduced by 84.31% (in ox-3), 90.45% (in ox-17) and 87.36% (in ox-28), respectively, and transcripts of *TAT* were decreased by 66.01% (in ox-3), 71.28% (in ox-17) and 68.50% (in ox-28), respectively. Yet no cases of ≥2-fold down- regulation were seen in *PAL*, *4CL1*, *4CL2* and *HPPR* (Fig. 7A, B). In contrast to overexpression of *SmMYB39*, silencing of this gene up-regulated expression levels of the six key enzyme genes. Relative to the empty-vector control lines, transcripts of *C4H* reached 9.46- (in RNAi-11), 7.71- (in RNAi-19) and 8.04-fold (in RNAi-25), respectively, and transcripts of *TAT* reached 6.67- (in RNAi-11), 5.75- (in RNAi-19) and 6.57-fold (in RNAi-25), respectively. Expression levels of *PAL*, *4CL1*, *4CL2* and *HPPR* were also enhanced, while none of them exhibited a ≥2- fold increase (Fig. 7C, D).

### *SmMYB39* inhibits C4H and TAT activities

**Figure 8:**
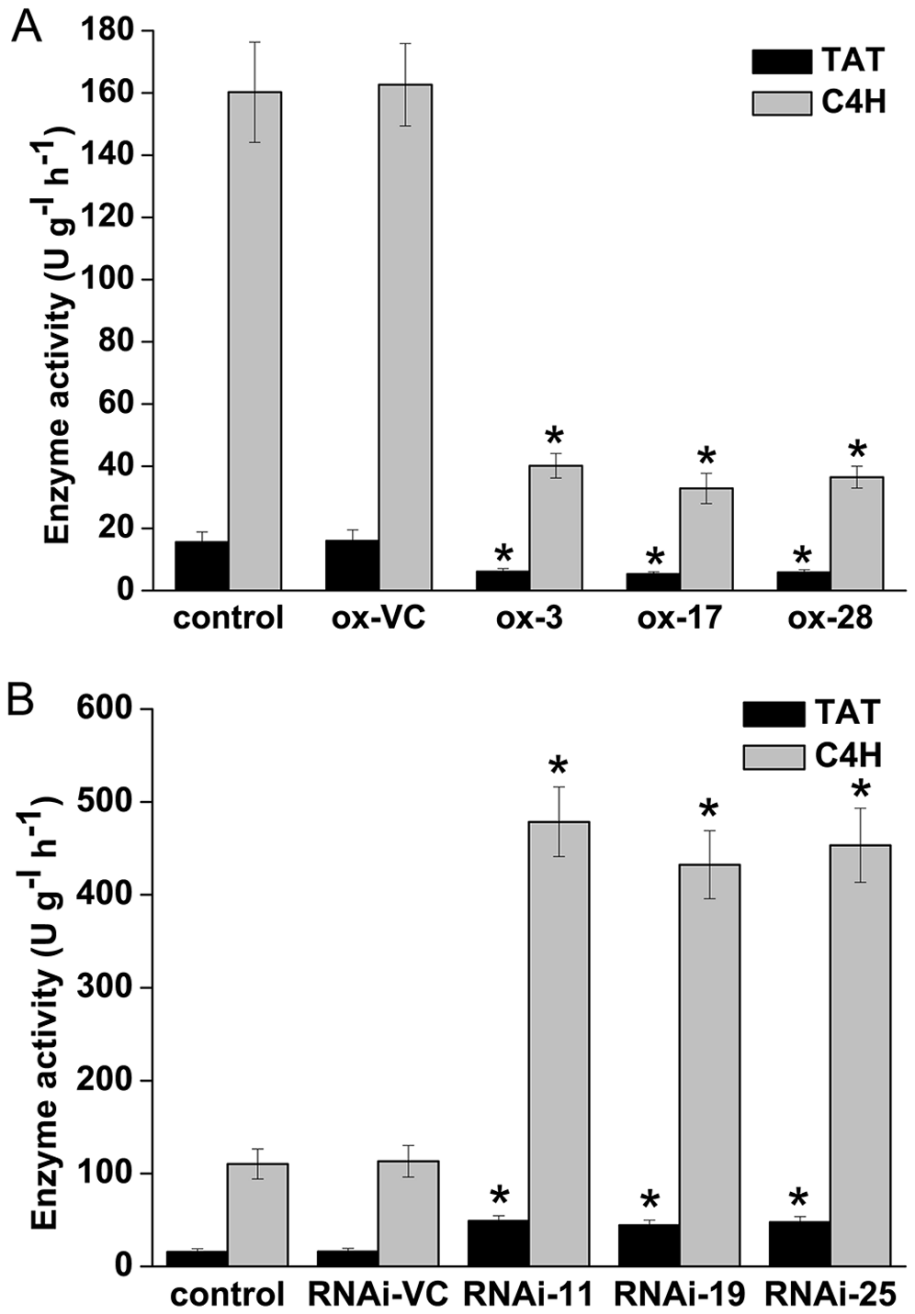
Enzyme activities analysis of C4H and TAT in transgenic lines and controls of *S. miltiorrhiza*. Data presented here are the mean of three replicates with error bars indicating ± SD. The asterisks indicate statistically significant differences (*P*<*0.05*) compared to the empty vector control. control: untransformed plant; ox-VC, RNAi-VC: empty vector controls of *SmMYB39*-overexpressing lines and *SmMYB39*-RNAi lines; ox-3, ox-17, ox-28: *SmMYB39*-overexpressing lines; RNAi-11, RNAi-19, RNAi-25: *SmMYB39*-RNAi lines; C4H: cinnamic acid 4-hydroxylase; TAT: tyrosine aminotransferase.

Activities of C4H and TAT, whose expression were regulated by *SmMYB39*, were determined in this study. As shown in Fig. 8, activities of these two enzymes were dramatically (*P*<0.05) reduced in the three *SmMYB39*-overexpressing lines. Compared with the empty-vector lines, activity of C4H was decreased by 75.31% (in ox-3), 79.79% (in ox-17) and 77.58% (in ox-28), respectively, and activity of TAT was down-regulated by 61.90% (in ox-3), 66.56% (in ox-17) and 63.52% (in ox-28), respectively (Fig. 8A). RNAi-mediated silencing of *SmMYB39* obviously (*P*<0.05) stimulated activities of C4H and TAT. The C4H activity in the *SmMYB39*-RNAi lines was approximately 4.23- (in RNAi-11), 3.82- (in RNAi-19) and 4.00- fold (in RNAi-25) of that in the empty-vector lines, respectively. TAT activity in these three transgenic lines reached about 3.06- (in RNAi-11), 2.77- (in RNAi-19) and 2.99-fold (in RNAi-25) relative to the empty-vector lines, respectively (Fig. 8B).

## Discussion

Although many subgroup 4 R2R3 MYB transcription factors have been identified as transcriptional repressors, the mechanism of repression is still not totally understood. As one of first R2R3 MYB transcription factors suggested as transcriptional repressors of phenylpropanoid pathway, AtMYB4 was reported to act both by directly repressing and by competing with activators on binding motifs located on the promoters of target genes [25]. Most of C-terminus of subgroup 4 R2R3 MYB protein sequences contain three typical protein motifs ‘LlsrGIDPxT/SHRxI/L’, ‘pdLNLD/ELxiG/S’ and‘CX_1–2_CX_7–12_CX_2_C’, which were termed as C1, C2 and Zf motif, respectively [22], [26], [45]. Amongst them the C2 motif was proposed to play key roles in repression activity [23], [25], [44]. All the three motifs were present in the C-terminus of SmMYB39 and its highly homologous protein sequences that have been reported to act as repressors of phenylpropanoid pathway (Fig. 2) [27], [44]–[46]. MYB proteins from different plant species with a similar sequence structure were suggested to regulate similar pathways and possess same type of regulation (activation or repression) [28]. The overall structural homology between SmMYB39 and MYB proteins mentioned above suggests that SmMYB39 function as a true repressor of RA pathway in *S. miltiorrhiza*.

R2R3 MYB transcription factors have been demonstrated to regulate different branches of phenylpropanoid metabolism in plants, such as biosynthesis of lignin [26], anthocyanin and flavonols [28]. Yet there are only a few reports about whether anther branch of phenylpropanoid metabolism, the RA biosynthetic pathway, can be regulated by R2R3 MYB transcription factors. For example, Zhang et al. (2010) revealed that ectopic expression of AtPAP1 from *Arabidopsis* strikingly induced the accumulation of SAB in transgenic *S. miltiorrhiza* [47]. In this study, Overexpresison/RNAi-mediated silencing of *SmMYB39* in *S. miltiorrhiza* plantlets dramatically (*P*<0.05) altered accumulations of 4-coumaric acid, rosmarinic acid, salvianolic acid B and salvianolic acid A, indicating that the RA pathway could be regulated by the R2R3 MYB transcription factor. Besides, content of *t*-cinnamic acid remained consistent in all the tested lines, implying that this precursor was not the bottleneck of RA biosynthesis.

A single R2R3 MYB transcription factor usually has several target genes and regulates multiple steps of the pathway that it is involved in [48]. This enables the regulator to control the metabolic flux more efficiently. In this study, we identified two key enzyme genes, *C4H* and *TAT*, whose transcripts and enzyme activities were all regulated by SmMYB39 as the target genes of this transcription factor. C4H (EC 1.14.13.11) is the second key enzyme in the phenylpropanoid pathway and catalyzes the hydroxylation of *t*-cinnamic acid to 4-coumaric acid [49]. *C4H* gene has been identified as the target gene of subgroup 4 R2R3 MYB transcription factors in many plant species, such as AmMYB308 and AmMYB330 from *Antirrhinum majus* [24], AtMYB4 from *Arabidopsis* [25], PhMYB4 from *Petunia* × *hybrida* [46] and VvMYB3 from grapevine [50]. By down-regulating of *C4H*, these transcription factors reduced biosynthesis of various phenylpropanoid metabolites. TAT (EC 2.6.1.5) is the first key enzyme of the tyrosine-derived pathway and transaminates tyrosine to 4-hydroxyphenylpyruvic acid [13]. *TAT* is involved in biosynthesis of several metabolites including rosmarinic acid, tocopherols and plastoquinones [13], [51], [52], while little is known about transcription factors that regulate this enzyme gene. C4H and TAT belong to the two parallel tributaries of RA pathway, phenylpropanoid pathway and tyrosine-derived pathway, respectively. With the co-ordinate control of the two key enzyme genes, SmMYB39 effectively regulated the accumulations of phenolic acids in *S. miltiorrhiza*.

Transcription factors usually active or repress gene transcripts by binding to cis-acting elements contained within promoters of target genes [53]. R2R3 MYB proteins from subgroup 4 have been reported to repress phenylpropanes metabolism by binding to AC elements [27], [44], [45], [54]. In this work, promoters of *C4H* gene (Genbank accession number: GQ896332.1) and *TAT* gene (Genbank accession number: EF192320.1) were analyzed with PlantCARE program (http://bioinformatics.psb.ugent.be/webtools/plantcare/html/). Results showed that both of them contained one AC-I elements with sequence ACCTACC (approximately 228 bp upstream from the transcription initiation site of *C4H* promoter and 776 bp upstream from the transcription initiation site of *TAT* promoter). SmMYB39 was likely to bind these AC elements and regulated the two parallel pathways of RA pathway simultaneously.

In summary, this work isolated and characterized a subgroup 4 R2R3 MYB transcription factor that regulated biosynthesis of phenolic acids in *S. miltiorrhiza*. It was located in nucleus and acted as a repressor by suppressing transcripts of *C4H* and *TAT*. Our results will be useful to better understand the regulating mechanism of phenolic acids production in *S. miltiorrhiza* and provide a train of thought to improve contents of bioactive compounds in this traditional herbal.

## Supporting Information

Figure S1 **The expression plasmids used in transformation and molecular analyses of transgenic** ***S. miltiorrhiza*** **plantlets.** (A) Schematic representation of transgene expression plasmids. *P_NOS_*, nopaline synthase promoter; *NPTII*, neomycin phosphotransferase II gene; *T_NOS_*, nopaline synthase terminator; *P_35S_*, CaMV 35S promoter; *F-259bp*, *R-259bp*, forward 259 bp and reverse 259 bp fragments of *SmMYB39*; *T_OCS_*, octopine synthase terminator; LB, T-DNA left border; RB, T-DNA right border; NPTII-1F, NPTII-1R, Sm35S1F, SmMYB39-11R, NPTII-2F, NPTII-2R, Sm35S2F, SmMYB39-22R, primers used for identification of transgenic lines; Restriction sites are marked. (B) PCR analyses for *SmMYB39*-overexpression and *SmMYB39*-RNAi *S. miltiorrhiza* plantlets. M, DNA marker (100–1200 bp); P, the corresponding engineered plasmids (positive control); N, the wild-type *S. miltiorrhiza* plantlet (negative control); C, no template control.
(TIF)

Figure S2 **Schematic representation of gene structure, predicted secondary structure and 3-D structure of SmMYB39.** (A) Structure of the *SmMYB39* genomic sequence. The exons are shown as blocks and the intron as line. The R2 and R3 repeats that constitute the MYB domain are shown as green and yellow shaded boxes, respectively. Numbers refer to position relative to the first nucleotide of the start codon. (B) Predicted secondary structure of SmMYB39 by SOPMA. Alpha helices, extended strands, beta turns and random coils are indicated by the longest, the second longest, the second shortest and the shortest vertical lines, respectively. (C) Predicted 3-D structure model of SmMYB39 MYB domain by SWISS-MODEL program. The structural model was based on the known crystal structure of a c-MYB from *Mus musculus* (PDB ID: 1H88, Chain ID: C) and shown using protein solid ribbon.
(TIF)

Text S1 **The prediction of secondary structure and 3-D structural model of SmMYB39.**
(DOCX)
