# Supplementary figures and images for: Cloning and Characterization of a Putative R2R3 MYB Transcriptional Repressor of the Rosmarinic Acid Biosynthetic Pathway from Salvia miltiorrhiza
Source: PLoS One. 2013 Sep 10;8(9):e73259. doi: 10.1371/journal.pone.0073259 (PMC3769309; doi:10.1371/journal.pone.0073259)

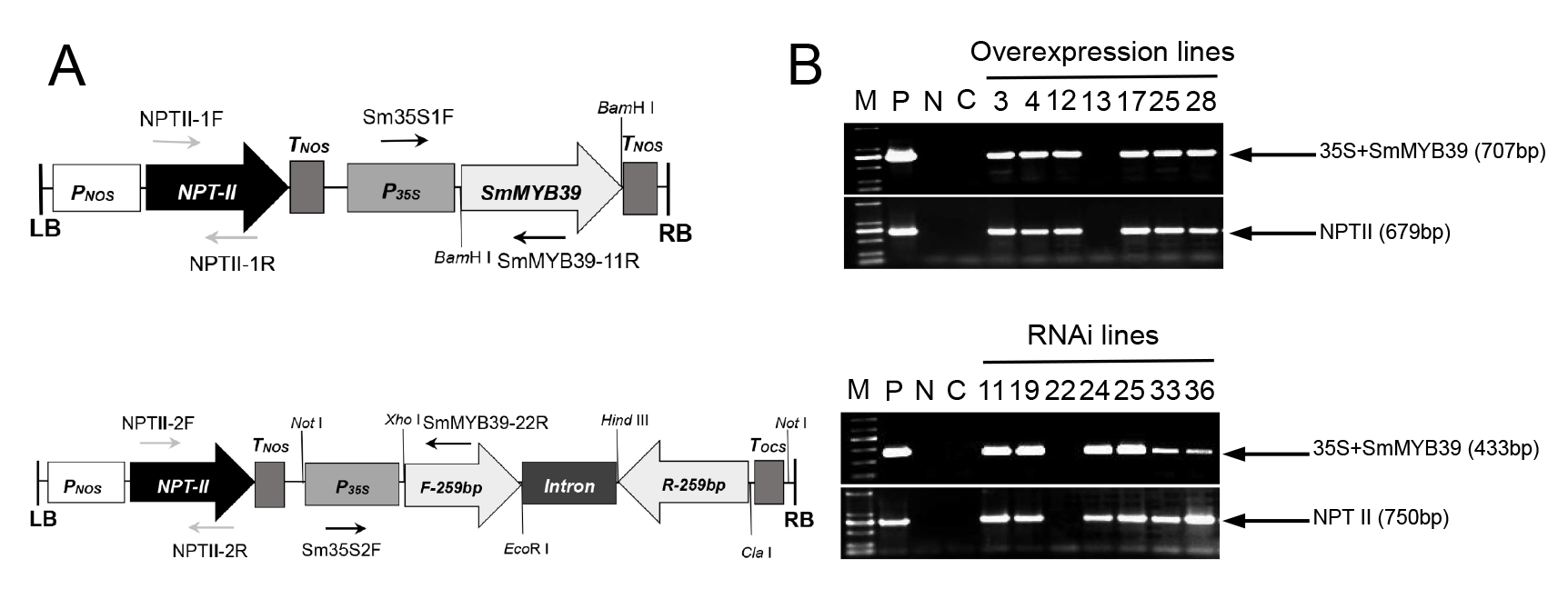

Supplement: Figure S1 — The expression plasmids used in transformation and molecular analyses of transgenic S. miltiorrhiza plantlets. (A) Schematic representation of transgene expression plasmids. PNOS, nopaline synthase promoter; NPTII, neomycin phosphotransferase II gene; TNOS, nopaline synthase terminator; P35S, CaMV 35S promoter; F-259bp, R-259bp, forward 259 bp and reverse 259 bp fragments of SmMYB39; TOCS, octopine synthase terminator; LB, T-DNA left border; RB, T-DNA right border; NPTII-1F, NPTII-1R, Sm35S1F, SmMYB39-11R, NPTII-2F, NPTII-2R, Sm35S2F, SmMYB39-22R, primers used for identification of transgenic lines; Restriction sites are marked. (B) PCR analyses for SmMYB39-overexpression and SmMYB39-RNAi S. miltiorrhiza plantlets. M, DNA marker (100–1200 bp); P, the corresponding engineered plasmids (positive control); N, the wild-type S. miltiorrhiza plantlet (negative control); C, no template control. (TIF) [file pone.0073259.s001.tif]

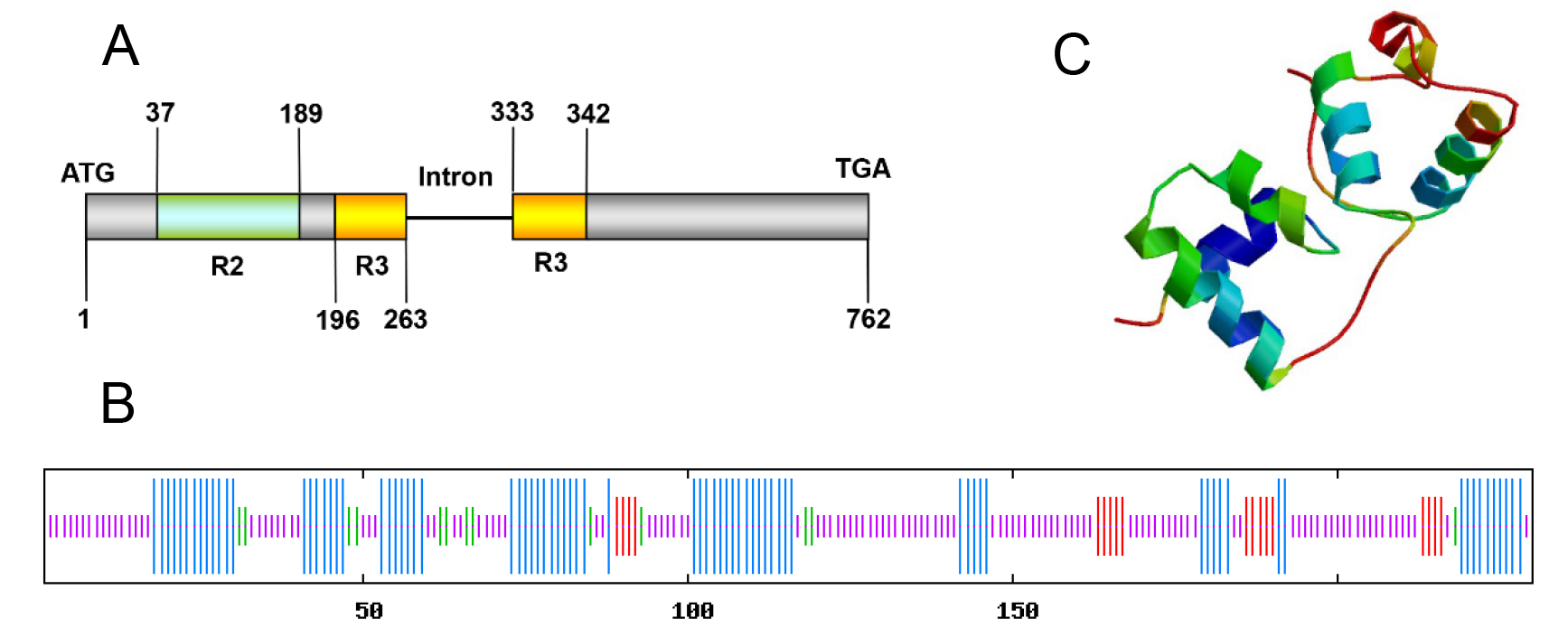

Supplement: Figure S2 — Schematic representation of gene structure, predicted secondary structure and 3-D structure of SmMYB39. (A) Structure of the SmMYB39 genomic sequence. The exons are shown as blocks and the intron as line. The R2 and R3 repeats that constitute the MYB domain are shown as green and yellow shaded boxes, respectively. Numbers refer to position relative to the first nucleotide of the start codon. (B) Predicted secondary structure of SmMYB39 by SOPMA. Alpha helices, extended strands, beta turns and random coils are indicated by the longest, the second longest, the second shortest and the shortest vertical lines, respectively. (C) Predicted 3-D structure model of SmMYB39 MYB domain by SWISS-MODEL program. The structural model was based on the known crystal structure of a c-MYB from Mus musculus (PDB ID: 1H88, Chain ID: C) and shown using protein solid ribbon. (TIF) [file pone.0073259.s002.tif]
