# Supplementary material for: Cloning and Characterization of a Putative R2R3 MYB Transcriptional Repressor of the Rosmarinic Acid Biosynthetic Pathway from Salvia miltiorrhiza
Source: PLoS One. 2013 Sep 10;8(9):e73259. doi: 10.1371/journal.pone.0073259 (PMC3769309; doi:10.1371/journal.pone.0073259)
Supplement: Text S1 — The prediction of secondary structure and 3-D structural model of SmMYB39. (DOCX) [file pone.0073259.s003.docx]

**Text S1: The prediction of secondary structure and 3-D structural model of SmMYB39**

The prediction of secondary structure by SOPMA [[1](#_ENREF_1)] indicated that the deduced SmMYB39 protein consisted of 33.91% of α-helices, 5.65% of β-turns, 7.83% of extended strands, and 52.61% of random coils (Fig. S2B). The 3-D structural model of SmMYB39 (Fig. S2C) contained two MYB-type HTH domains, which have been suggested to bind directly to the DNA major groove [[2](#_ENREF_2)].

**References**

1. Combet C, Blanchet C, Geourjon C, Deleage G (2000) NPS@: Network Protein Sequence Analysis. Trends Biochem Sci 25: 147-150.

2. Stracke R, Werber M, Weisshaar B (2001) The *R2R3-MYB* gene family in *Arabidopsis thaliana*. Curr Opin Plant Biol 4: 447-456.
